# A new device for continuous assessment of gut perfusion: proof of concept on a porcine model of septic shock

**DOI:** 10.1186/cc13992

**Published:** 2014-07-16

**Authors:** Matthias Jacquet-Lagrèze, Jeanne-Marie Bonnet-Garin, Bernard Allaouchiche, Olivia Vassal, Damien Restagno, Christian Paquet, Jean-Yves Ayoub, Jérôme Etienne, François Vandenesch, Olivier Daulwader, Stéphane Junot

**Affiliations:** 1 Service d’Anesthésie-Réanimation, Hospices Civils de Lyon, hôpital Edouard-Herriot, 5 place d’Arsonval, 69437 Lyon, Cedex 03, France; 2 Université de Lyon, EA 4174 Hémostase, Inflammation et Sepsis, VetAgro Sup - Campus Vétérinaire de Lyon, 1 Avenue Bourgelat, 69280 Marcy l'Étoile, France; 3 Université Claude Bernard, 43 Boulevard du 11 Novembre 1918, 69100 Villeurbanne, Lyon, France; 4 Laboratoire de Microbiologie, Groupement Hospitalier Est, Lyon, France

## Abstract

**Introduction:**

We evaluate an innovative device consisting of an enteral feeding tube equipped with a photoplethysmography (PPG) sensor in contact with the duodenal mucosa. This study aims to determine if the PPG signal, composed of a continuous (PDC) and a pulsatile part (PAC), is a reliable method to assess gut perfusion in a porcine model of septic shock.

**Method:**

Fourteen piglets were anesthetized and mechanically ventilated. They were randomly assigned to two groups: the nonseptic (NS) group received an infusion of Ringer’s lactate solution (RL) alone, the septic (S) group received in addition a suspension of live *Pseudomonas aeruginosa.* Heart rate (HR), pulse oximetry (SpO_2_), mean arterial pressure (MAP), cardiac index (CI) and serum lactates were recorded and gut microcirculation (GM) was monitored with a laser Doppler probe applied on the duodenal serosa. PDC and PAC were given by the PPG probe inserted in the duodenum. Data was collected every 15 minutes (t_0_, t_15_…) during 150 minutes (t_150_). After administration of the bacteria suspension (t_0_), resuscitation maneuvers were performed following a defined algorithm. GM PAC, and PDC were expressed as variation from baseline (GM_var_, PAC_var_, PDC_var_). Analysis of variance (ANOVA) with repeated measures was performed to compare hemodynamic variables, with Bonferroni correction as *post hoc* analysis on t_0_, t_60_ and t_150_.

**Results:**

One piglet was withdrawn from analysis due to a defective probe. S group (six piglets) received resuscitation therapy while NS group (seven piglets) did not. A significant group effect was found for the all parameters except HR. *Post hoc* analysis found a significant decrease for GM and PAC at t_60_. The correlation between PAC, PDC and microcirculatory parameters were as follows: r_PACvar-GMvar_ = 0.496, *P* <0.001, r_PDCvar-GMvar_ = 0.244; *P* = 0.002. In the septic group, correlations were as follows: r_PAC-lactate_ = -0.772, *P* <0.001; r_PDC-lactate_ = -0.681, *P* <0.01). At the onset of shock, a decrease of PAC, PDC and GM occurred before the alteration of MAP.

**Conclusions:**

PAC and PDC decreased at the onset of shock and were correlated with GM and lactate. These results confirm that PPG signal reliably reflects the early perfusion alteration of the gut. Further studies should assess the clinical use of this device.

## Introduction

Gut has been widely described as the motor of multiple organ dysfunction syndrome during sepsis [1]. Therapy guided by macrocirculatory parameters such as mean arterial pressure (MAP), central venous pressure and cardiac output (CO) are uncoupled with microcirculation in sepsis [2] and may fail to improve microcirculation [3]. Moreover, early correction of microcirculatory disorder is linked with improved survival [4]. Ability to monitor microcirculation may serve as a guide for resuscitation in sepsis and improve treatment of this condition. [5]. So far, regional perfusion and microcirculation has been evaluated in the sublingual area, as it is readily accessible in the awake and sedated patient. Alteration of the perfusion of sublingual capillaries is an accurate sign of severity and a good predictor of poor outcome in patients with sepsis [6]. However, it may fail to detect early alteration of gut microcirculation [7]. Thus, a monitoring device capable of evaluating gut perfusion would be of great interest for critically ill patients [8]. Among the technologies available to monitor tissue perfusion, photoplethysmography (PPG) is an optical measurement technique widely used to assess pulse oximetry and has been described for evaluation of skin perfusion [9,10]. It is linked to macro- and microcirculatory changes. It has been used successfully to assess gut perfusion in different clinical contexts [11,12]. To the best of our knowledge, it has not been evaluated during sepsis. Compared to existing gastric laser Doppler (LD) probes PPG technology is less expensive and may be less sensitive to motion artifact [10]. PPG is a noninvasive technique that measures relative blood volume changes in the blood vessels close to the sensor [9]. PPG signal can be divided into two parts: the pulsatile component of the PPG, 'AC’ or PAC, dependent on heart rate (HR), is superimposed on a large component called the 'DC’ or PDC and is thought to be influenced by the tissue properties and their blood content.

As there is no validated bedside technology available to score microvascular perfusion of the gut in real time, in the present study, we propose to evaluate a new tool to assess gut perfusion [13]. This innovating device (APD Advanced Perfusion Diagnostics, Lyon, France) consists of a PPG sensor fixed on a balloon at the tip of an enteral feeding tube. This noninvasive device allows a contact between the probe and the duodenal mucosa and gives real-time and continuous measurements of two signals, PAC and PDC. This study aims to determine if the PPG signal measured in the duodenum is a reliable method to assess microcirculatory impairment in a porcine model of septic shock.

## Materials and methods

Animals were used according to ethical rules supervised by the ethical committee of VetAgroSup (agreement no. 1252) and European regulation (Directive EU 86/609).

### The photoplethysmographic probe

**Figure 1:**
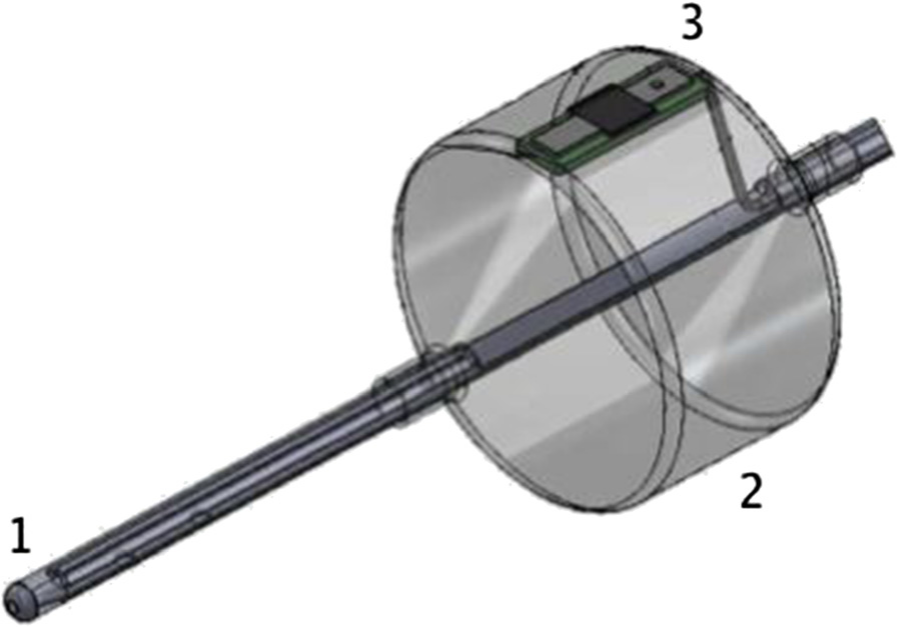
**Tip of the duodenal photoplethysmographic probe. (1)** An enteral feeding tube **(2)** with a balloon. **(3)** A photoplethysmographic sensor.

**Figure 2:**
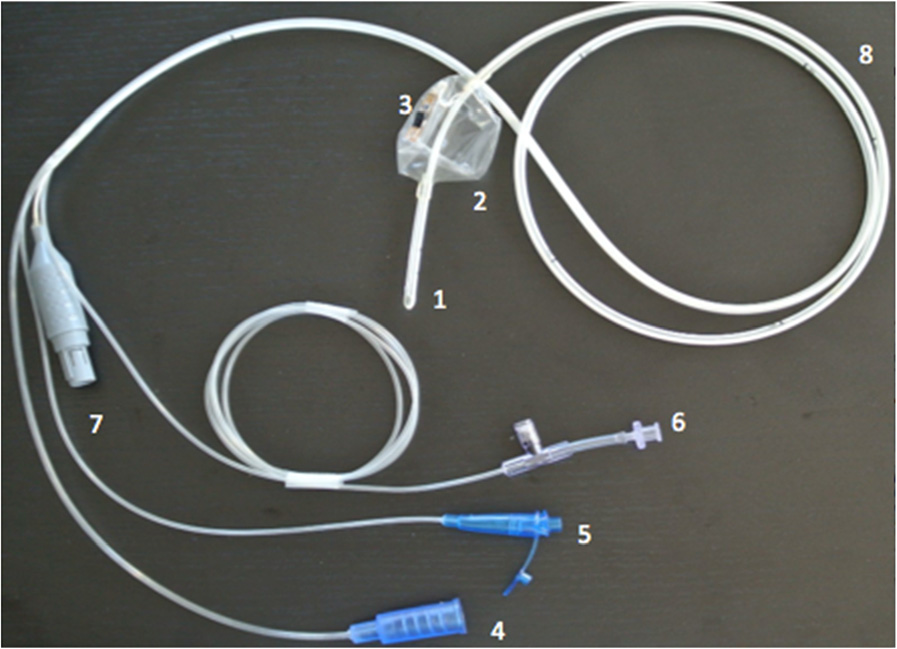
**The duodenal photoplethysmographic probe.** The duodenal feeding tube with the photoplethysmographic probe. **(1)** Enteral feeding tube tip for post-pyloric nutrition. **(2)** Inflatable balloon. **(3)** Photoplethysmographic sensor. **(4)** Oro- or nasojejunal feeding line. **(5)** Aspiration line. **(6)** Line dedicated to inflate and deflate the balloon and to measure intra-abdominal pressure. **(7)** Connection line to the sensor. **(8)** Aspiration hole.

The probe consists of a 75 cm long feeding tube with a balloon that contains the photoplethysmographic sensor constituted of an infrared emitter and receptor (Figure 1). It is designed to be inserted in human patients through the mouth or the nose, as any enteral feeding tube (Figure 2). The balloon is positioned after the pylorus with the tip of the feeding tube in the duodenum. The proper placement of the tube can be confirmed with X-ray.

The device contains several lumens. One lumen is dedicated to the inflation and deflation of the balloon so that the contact between the intestinal mucosa and the sensor is adequate. Another lumen allows aspiration of the gastric content, while a feeding lumen permits delivering of enteral feeding within the duodenum. The system is equipped with a pressure sensor that allows intra-abdominal pressure measurement and inflation of the balloon according to a given transmural pressure (set at 2 kPa). In our experimental study, the balloon was inflated for five minutes every six minutes. The PPG sensor is composed of a light-emitting diode 12 mm distant from a photodiode, the wavelength of light emitted is 905 nm in the infrared spectrum: the PPG signal can be decomposed into one continuous part (PDC) and one pulsatile part (PAC). The signal processing is performed so that any noise disturbing the measurements is removed by a passband Butterworth filter. Cutoff frequency for separating the AC part from the DC part has been set at 1 Hz for the third-order Butterworth filter. The filter aims to dampen respiratory harmonics, duodenum peristalsis, and electromagnetic disturbances. The area under the curve of the pulsatile part of the signal reflects the pulsatile flow (PAC), which is given in arbitrary units (AU). PDC is the area under the PPG signal minus the PAC given in AUs.

### Animals

Fourteen healthy female piglets weighing between 25 and 40 kg and two to three months old were allowed to acclimatize for a minimum of one week before the experiment. They were fed with standard food, and had access to water at all time. Twelve hours before the beginning of the experimental phase, solid food was withdrawn while access to water was still allowed.

### Study design

The piglets were randomly assigned to two groups: one septic, one control. Animals of the control group (n = 7) received an infusion of Ringer’s lactate solution (RL) alone; those of the sepsis group (n = 7) received in addition a suspension of live *Pseudomonas aeruginosa* (5 × 10^8^ colony-forming units (CFUs)/mL perfused at 0.3 mL/20 kg/min).

#### Anesthesia and ventilation

Animals were sedated with an intramuscular administration of a 1:1 mixture of tiletamine and zolazepam (Zoletil™ 100, 100 mg/mL, Virbac, Carros, France), 3.0 mg/kg and morphine 0.2 mg/kg. Induction was carried out with propofol (Propovet, 10 mg/mL, Axience, Pantin, France) 4.0 mg/kg intravenously. Maintenance of anesthesia was ensured by volatile anesthesia with isoflurane (Vetflurane, Virbac, Carros France) given to effect using a 50% fraction of inspired oxygen and with morphine (0.1 mg/kg/4 h). After induction of anesthesia, animals were orotracheally intubated and placed under mechanical ventilation with controlled tidal volume set at 8.0 mL/kg and a respiratory rate of 12 breaths/min. Intravenous RL was administered during the experiment at a basal rate of 10 mL/kg/h.

#### Equipment

Pulse oximetry and electrocardiogram were used to continuously measure peripheral oxygen saturation (SpO2) and HR.

A right and a left lateral cervicotomy were performed to isolate the jugular vein and the carotid artery. An arterial catheter was inserted into the left carotid artery for continuous assessment of the MAP and blood sampling. A central venous catheter was inserted in the left jugular vein for fluid and drug administration. A pulmonary arterial catheter (Edwards Lifesciences™, Irvine, CA, USA) was inserted in the right jugular vein and advanced into the pulmonary artery for measurement of mean pulmonary artery pressure (MPAP, mmHg), pulmonary capillary wedge pressure (PCWP, mm Hg) and cardiac output (CO, L/min). The appropriate location of the catheter tip was determined by direct visualization of the pressure waveform. Cardiac index (CI, L/min/m^2^) was calculated as the CO (L/min) divided by the body surface area (m^2^). Surface area was calculated as previously described [14]. Blood samples were analyzed with a blood gas analyzer (I-STAT System, Abbott, Chicago, IL, USA). Following a median laparotomy and a gastrotomy, the PPG device was inserted in the duodenum. A LD probe was positioned on the serosa of the intestinal wall, a few centimeters forward from the PPG probe cuff. It recorded a signal of microcirculation named gut microcirculation (GM) signal. The abdomen was then sutured closed (PDS II, Ethicon, Issy-les-Moulineaux, France) for the rest of the experiment.

### Bacterial preparation

A *Pseudomonas aeruginosa* reference strain (ATCC 27853) was used to induce septic shock. This strain was kept in a glycerolized heart/brain broth at -80°C at the microbiology laboratory (laboratoire de Bactériologie du Centre de Biologie et Pathologie Est, Hospices Civils de Lyon, Lyon, France). Forty-eight hours before the experiment, two subcultures were systematically performed to avoid any variation of the reference stain. Briefly, *P. aeruginosa* strain was defrosted and incubated at 37°C for 6 h in heart/brain broth (AES/BioMérieux, Marcy L’Etoile, France). Then, the broth was reseeded on blood agar plate media (BioMérieux, Marcy L’Etoile, France) and cultivated for 18 to 24 h at 37°C. Then, 50 mL of a bacterial suspension was prepared in sterile saline solution (Aguettant, Lyon, France) with a bacterial density between 1 and 5 × 10^8^ CFU/mL (density of one on the McFarland scale, API Densitometer, BioMérieux, Marcy L’Etoile, France) on the day of the investigation. The suspension was kept in a refrigerator ready for use less than two hours before infusion. To check the inoculum concentration, two dilutions of bacteria suspension were streaked on Mueller-Hinton agar plates by Spiral instrument (AES/BioMérieux, Marcy l’Etoile, France). These plates were read after overnight incubation at 37°C and compared to the abacus of the Spiral™ system to obtain concentration of bacteria suspension.

## Protocol and data collection

### Hemodynamics

#### Macrocirculation

Hemodynamic parameters were collected. Every pressure was measured with a reference level at the mid-chest. MAP and MPAP, HR and SpO2 were continuously recorded, while PCWP and CO was measured every 15 minutes. CO was assessed by thermodilution with two consecutive administrations of 10 mL of cold saline.

#### Microcirculation and biochemical analysis

GM and PPG were continuously recorded, blood samplings were carried out every 15 minutes to measure blood gas and plasma lactate.

#### Study design

After a 30-minute stabilization period, the animals were observed during a 30-minute baseline period, followed by one of the treatments described below. Parameters were recorded before (t_0_) every 15 minutes until the end of the experiment at t_150_. The animals were randomly assigned into two groups:

• Nonseptic group (NS group): seven piglets received an infusion of NaCl 0.9% at H_0_,

• Septic group (S group): seven piglets received an infusion of live *P. aeruginosa* (5 × 10^8^ CFU/ml perfused at 0.3 ml/20 kg/min).

The systolic pulmonary arterial pressure (SPAP) was monitored. If SPAP reached 45 mmHg, bacterial infusion was stopped to limit the right ventricular afterload in order to obtain a reproducible hyperdynamic state without any right heart dysfunction, as previously described in this model of septic shock [15,16].

### Resuscitation maneuvers algorithm

A decrease of 50% of the basal CO or MAP was the triggering factor for the start of resuscitation maneuvers. The goal was to maintain MAP and CO above this percentage. Norepinephrine was administered if the MAP decreased more than 50% of its basal value. Initial dose was 0.02 μg/kg/min. Fluid boli consisted of 250 mL of RL and were given in case of hypotension or a decrease of CO under 50% of its basal value. Fluid boli were continued until no fluid responsiveness was observed. Fluid responsiveness was defined as an increase of the cardiac output of 15% after fluid challenge. If CO remained low with no fluid responsiveness, dobutamine was started. An initial dose rate of 5 μg/kg/min of dobutamine was administered and increased up to 20 μg/kg/min if CO remained low.

### Biological samples and euthanasia

Just before the piglet euthanasia (T61™, Intervet, Beaucouzé, France) three hours after t_0_, at t_180_, biopsies of the duodenum mucosa in contact with the probe and in another place nearby were performed. Histology was performed (laboratoire d’anatomopathologie, VetAgro Sup, Marcy, France) to determine whether the tip of the probe or its inflated balloon could injure duodenal mucosa.

### Statistical analysis

Statistical analysis was performed with R software [17]. We used several package of the CRAN R project and computed descriptive statistics for all the data [18]. We used a Kolmogorov-Smirnov test to check the normality of variables distribution. In case of non-normality, data were expressed as median (25^th^ to 75th percentiles). Data were expressed as variation of the mean of the three measures made at baseline (t_baseline_ = t_-30,_ t_-15_ and t_0_), already described for LD [19]. We calculated the coefficient of variation of the data at baseline on the normalized value for all the piglets; this was calculated as the standard deviation divided by the mean. Hemodynamic data as GM and PAC were divided by the mean of the three baseline values and expressed as variations (GM_var_, PAC_var_ and PDC_var_). We used ANOVA with repeated measures to detect if there was an effect of time and group on the different hemodynamic parameters. Our data were not normally distributed, but we checked the more or less normal distribution on the frequency histogram, as ANOVA is not very sensitive to moderate deviations from normality [20-22]. A Mann-Whitney *U* test and Wilcoxon test were performed as appropriate with the Bonferroni correction to assess respectively the difference between groups at different times and to compare it to baseline. As the data were mainly not normally distributed, we evaluated relationships between hemodynamic variables, microcirculatory variables and lactates using Spearman’s correlation. We observed a great variability of duration between bacterial injection and occurrence of shock so we also performed analysis at the onset of shock for each piglet. In order to evaluate if PPG alteration preceded macrocirculatory impairment, three times were defined as follow: t_0_ was the same t_0_, time before shock (t_bs_) which was 20 minutes before the third point defined as the time before resuscitation (t_br_). T_br_ was defined as the time MAP decreased to under 40 mmHg. Statistical significance was defined as a *P* value lower than 0.05.

## Results

**Figure 3:**
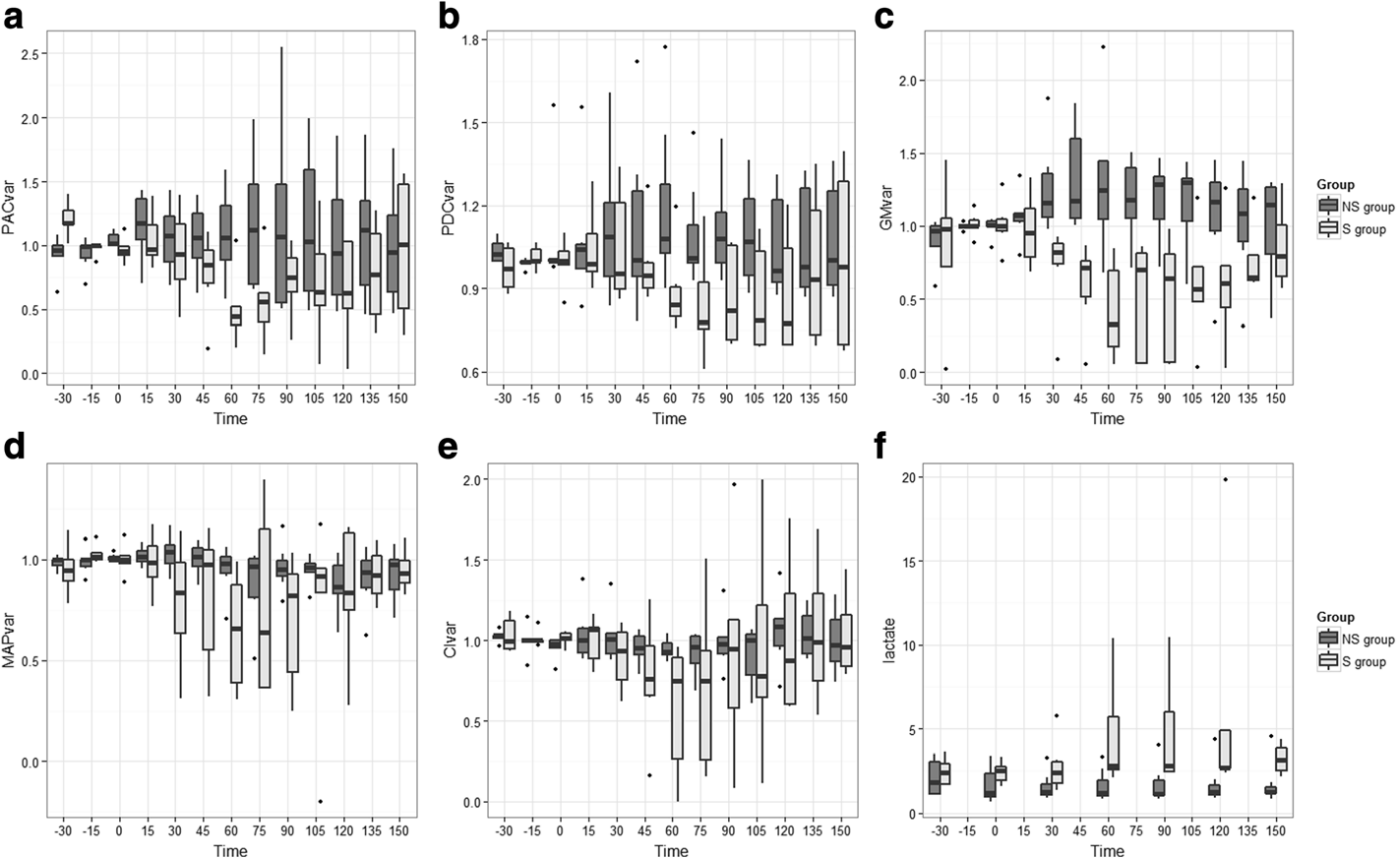
**Evolution of the hemodynamic parameters. (a)** PAC_var_, variation of the component AC of the photoplethysmographic signal, **(b)** PDC_var_, variation of the component DC of the photoplethysmographic signal, **(c)** GM_var_, variation of the laser Doppler signal on the serosa of the duodenum, **(d)** MAP_var_, variation of the mean arterial pressure, **(e)** CI_var_, variation of the cardiac index. T_0_ is the time of bacterial injection in the septic group (S group) and placebo for the nonseptic group (NS group). T_150_ is 150 minutes after bacterial injection. ANOVA were performed for each parameter: GM, group effect *P* <0.001; PAC, group effect *P* = 0.035; PDC, group effect *P* <0.001; MAP, group effect *P* <0.001, time effect *P* = 0.015; CI, group effect *P* <0.001; heart rate (HR), group effect *P* <0.093; lactate group effect *P* <0.001.

Fourteen piglets weighing 30 kg [28,35] aged three months were studied, one piglet was withdrawn from the analysis due to a defect in the PPG probe. One of the tested probes was found to be defective due to a contact failure with the light-emitting diode. Bacterial strains were verified and no concentration difference was found. Hemodynamic data were tested at baseline and no significant difference was found. The main characteristics of the piglets at the end of the experimentation are detailed in Table 1. As one can see on the Figures 3a and b, GM, PAC and PDC parameters were stable at baseline, coefficient of variation for PAC, PDC and GM were respectively 14%, 11% and 23%.

**Table 1:** **Main characteristics of the piglets in the septic and nonseptic group at t**_**150**_

	**Septic median [IQR]**	**Nonseptic median [IQR]**	***P *** **value**
Blood gas			
**pH**	7.35 [7.34; 7.38]	7.41 [7.37; 7.45]	0.09
**PaCO2 (mmHg)**	51 [45; 55]	46 [41; 55]	0.71
**PaO2 (mmHg)**	265 [214; 292]	283 [263; 307]	0.38
**BE (mmHg)**	4.5 [0.6; 5.4]	6.7 [4.3; 8.3]	0.27
**PaO2/Fi02 (mmHg)**	364 [279; 390]	555 [516; 596]	<0.01
Resuscitation			
**Norepinephrine (μg)**	72 [18; 171]	0 [0; 0]	0.17
**Dobutamine (μg)**	0 [0; 4179]	0 [0; 0]	0.14
**Volume expansion (mL)**	785 [250; 1000]	0 [0; 0]	0.11
Outcome			
**Death before experimental end**	2	0	0.2

Results are expressed as median and interquartile range 25^th^ to 75^th^ percentile [IQR]. T_150_, 150 minutes after bacterial injection; PaCO2, carbon dioxide arterial partial pressure, PaO2, oxygen arterial partial pressure; FiO2, fraction of inspired oxygen.

### Resuscitation

Supplemental fluids were administered in the S group according to the predefined algorithm. Piglets received 750 [250; 1000] mL in the S group and no additional fluid was administered in the NS group. Total dobutamine and norepinephrine infusion at the end of the experiment was 0 [0; 5625] μg and 72 [18; 171] μg respectively in the S group. Neither dobutamine nor norepinephrine was administered in the NS group.

### Evolution of the PPG signal and other hemodynamic variables in both groups

A significant group effect was found for the GM, PAC, PDC, lactate (ANOVA with repeated measure) and MAP whereas no effect was found for CI or HR. (Figure 3a, b, c, for microcirculation, Figure 3d, e for macrocirculation parameters and 3 F for lactate). Tested at baseline, t_60_ and t_150_ we did not find any significant difference except for intergroup difference for GM and PAC at t_60_.

We found a significant intergroup difference for GM and PAC at t_60_. No significant intragroup difference was found in comparison with baseline when time effect was found with ANOVA.

### Correlation between PPG and other hemodynamic parameters for all data

**Figure 4:**
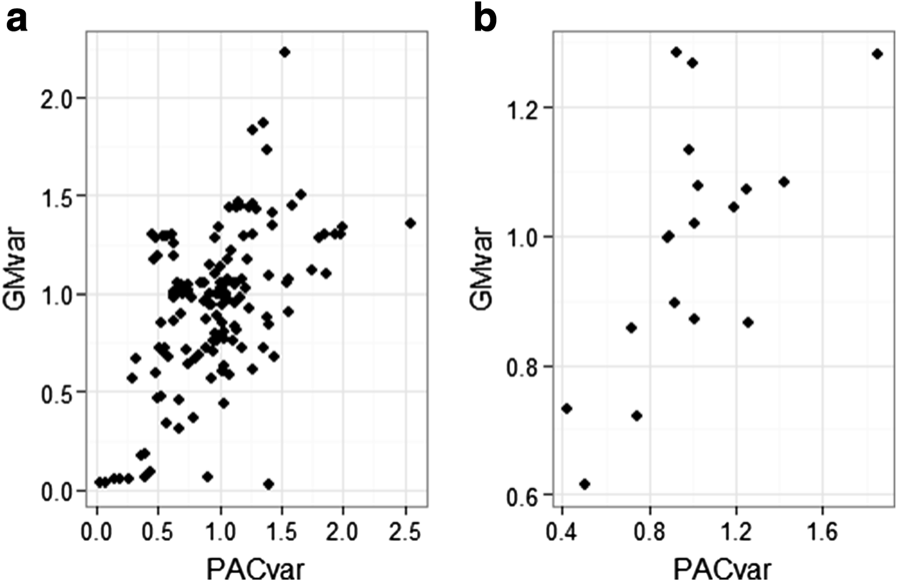
**Scatter plot of the GM**_**var **_**and PAC**_**var **_**in the whole experience and at the onset of shock.** PAC_var_, variation of the component AC of the photoplethysmographic signal, GM_var_, variation of the laser Doppler signal. **(a)** Whole experience. **(b)** Onset of shock.

The correlation between PAC, PDC and microcirculatory parameters during all the experiments in both groups were as follow: r_PACvar-GMvar_ = 0.496, *P* <0.001; r_PDCvar-GMvar_ = 0.244, *P* = 0.002. Correlations between PAC, PDC and macrocirculatory parameters (HR, MAP and CI) were not significant (Figure 4a).

### Correlation between hemodynamic parameters and lactate

In the S group lactate increased. Correlations with lactate in that group were as follow: r_PAC-lactate_ = -0.772, *P* <0.001; r_PDC-lactate_ = -0.681, *P* <0.001; r_GM-lactate_ = -0.112, *P* = 0.509. No significant correlations were found between CI, MAP, HR and lactate, PAC and PDC were the only parameters strongly correlated to lactate.

### Evolution of the PPG signal variation, GM variation and MAP variation at the onset of shock

**Figure 5:**
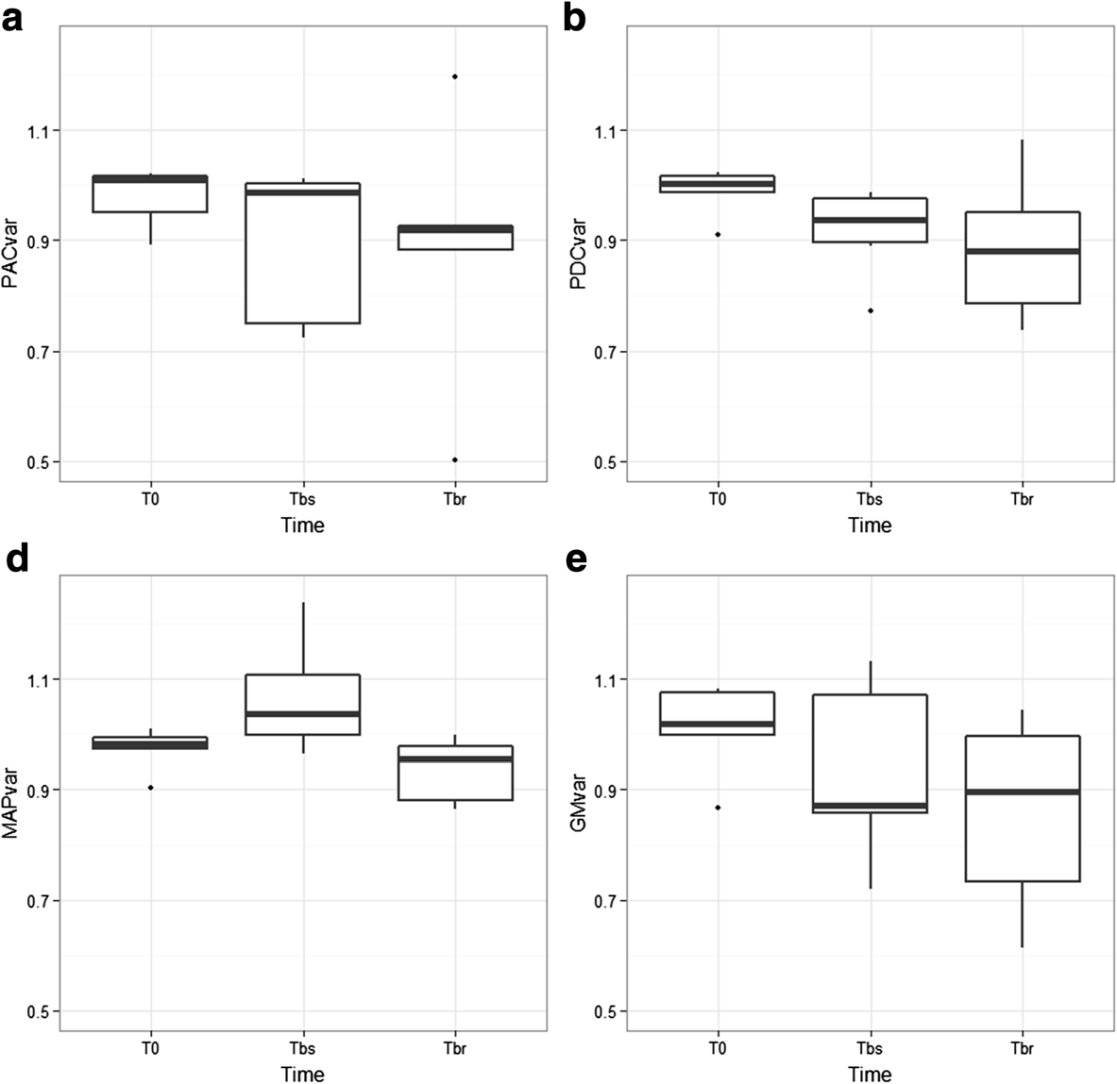
**Evolution of the hemodynamic parameters at the onset of shock in the septic group before resuscitation therapy. (a)** PAC_var_, variation of the component AC of the photoplethysmographic signal **(b)** PDC_var_, variation of the component DC of the photoplethysmographic signal, **(c)** MAP_var_, variation of the mean arterial pressure, **(d)** GM_var_, variation of the laser Doppler signal on the serosa of the duodenum. T_0_ is the time of bacterial injection in the septic group (S group) and placebo for the nonseptic group (NS group). T_br_, time before resuscitation is the time for each piglet when MAP decreased to 40 mmHg, which was defined as the shock in our piglet model and triggered resuscitation. T_bs_, time before shock is the time exactly 20 minutes before T_br_. ANOVA were performed for each parameter: GM, time effect *P* = 0.236; PAC, time effect *P* = 0.067; PDC, time effect *P* = 0.177; MAP, time effect *P* = 0.57. The evolution of the PDC and PAC is characterized by a near significant decrease that precedes the decrease of MAP.

The results are illustrated in (Figure 5a, b, c, d). The decrease of the PAC and PDC preceded the decrease of the MAP. The period between bacterial injection and the onset of shock lasted between 26 and 113 minutes, correlations were as follow: r_PACvar-GMvar_ =0.657, *P* = 0.003; r_PDCvar-GMvar_ = 0.527, *P* = 0.025. There was no correlation between MAP and PAC, PDC or GM) (Figure 4b).

### Safety of the probe

Histology of the duodenum samples (in contact with the probe versus at a distance) revealed no ischemic lesions and no difference between the sample sites.

## Discussion

The key findings of this experiment were a significant decrease of PAC and PDC during sepsis and a significant correlation with GM. PPG signal evolution was partially uncoupled with macrocirculatory parameters. A strong correlation between PAC, PDC and lactate concentration was found. Sepsis is characterized by a disruption in normal regulatory mechanisms that balance pro- and anti-inflammation and pro- and anti-coagulation. This imbalance results in cardiac and endothelial dysfunction, leading to macro- and microcirculation impairments. The GM is thought to be affected earlier than other territories [5], making its monitoring a promising means to improve treatment of sepsis. Improving gut perfusion has been associated with an improvement of intramucosal pH [23] and gastric mucosal tonometry, a surrogate of gastric mucosal perfusion [24], is one of the rare monitoring devices able to improve mortality of patients in septic shock [5]. In the aforementioned context, monitoring gut perfusion or microcirculation during sepsis is meaningful. Several methods can be used to assess microcirculation, including downstream markers such as tonometry, capnometry, microdialysis, biomarkers such as lactate and direct assessment tools such as LD or video microscopy (orthogonal polarization spectral and sidestream darkfield imaging techniques) [19]. LD has been widely used to assess gut perfusion in different clinical settings [25], surgeries [26,27] or sepsis [28]. Photoplethysmography is an optical method that has already been used for other medical applications such as measurement of oxygen saturation, evaluation of blood pressure and cardiac output, and assessment of autonomic function [9]. PPG has been compared to LD for the assessment of microcirculation of the skin [10] PPG has also been compared successfully with LD in the intestinal area to detect partial and total colonic ischemia. Both oximetry and PPG wave amplitude were used [29-31]. PPG has been tested to assess gut perfusion during surgery [12]. PPG and LD were not linked in some settings [32].

In our settings, the PPG signal appeared consistent and reliable during the experiment. The coefficient of variation at baseline was quite low with regard to the GM and to the change observed during septic shock of more than 50 and 20% for PAC and PDC, respectively.

When comparing PPG with GM, we must emphasize that both parameters do not measure the same signal. LD evaluates the speed of the erythrocytes composed of a continuous and a pulsatile flow, the second being due to acceleration of the flow during systole. PPG is based on light absorption at a specific length wave; the light absorption is proportional to hemoglobin tissue content [31]. The variation of the PAC, the GM and the PDC was similar during the experimental time with a steep decrease at the onset of sepsis followed after resuscitation maneuvers by a more gentle slope up, that barely reached the initial value of the signal. The evolution of the PAC, the PDC and the GM was explained by a significant effect of the septic status. The same effect was significant for MAP, CI and lactate but only PAC and GM were different in *post hoc* analysis at t_60_ making this parameter of particular interest.

PAC was significantly correlated with GM, confirming that this parameter can be used to evaluate microcirculation, even in critical conditions such as septic shock. The strong correlation between PAC and GM at the onset of sepsis found in our experimental condition is interesting as LD is considered a gold-standard method to assess GM [19]. This is consistent with previous studies comparing PPG and LD. Even if the two measures were not equivalent, a good correlation was found between LD and PPG in different conditions such as inspiratory-induced vasoconstrictive episodes [32] or wound healing [33], but its use and reliability in critical conditions was not tested. Some studies have concluded that finger PPG signal was inappropriate due to the poor signal obtained [34] while others stated that this method could have an interest. Several phenomena may affect the quality of the PPG signal, such as poor tissue perfusion, ambient light and motion artifact.

The correlation between PAC and GM was functionally confirmed by a correlation between PAC and lactate. The correlation with lactate was strong in the sepsis group and surprisingly this link was not found between the GM and lactate, presumably due to the small sampling size of our experimental setting. PAC and PDC had the strongest correlation with lactate while no macrocirculatory parameters such as CI, MAP or HR had a significant link with lactate. These results are noteworthy as lactate assay can predict death during sepsis and lactatemia is correlated to the number of failing organs [35]. Recently, early goal-directed therapy based on lactate measurement has yielded a decreased mortality in a subgroup of patients [36]. Besides, lactate assays have been shown to be equally efficient as mixed venous oxygen saturation monitoring [37] and a significant link between lactate and microcirculation has been established in previous studies [38]. Nevertheless, even if lactate is correlated to the severity of shock, it does not appear as a direct mirror of microcirculation or perfusion because of the multiple etiologies that can cause its increase [39]. One of the mechanisms thought to explain lactate increase is splanchnic hypoperfusion, leading to decreased lactate clearance [40,41] and explaining the link between lactate and gut perfusion assessment.

Another point is that there is no significant correlation between macrocirculatory parameters and the PPG signal. This is consistent with the concept largely described that microcirculation and macrocirculation are uncoupled during sepsis [2].

Similarly, the individual response to sepsis, the onset of shock and the duration of hemodynamic recovery after resuscitation is quite different between piglets. This asynchrony erased the evolution of the micro- and macrocirculation and resulted in a large distribution of value. For that reason, we performed an analysis centered on a point just before resuscitation. The decrease of the PAC, PDC and GM occurred earlier than the MAP. Over that period the correlation between PAC, PDC and GM was strong and significant, confirming an uncoupling between macrocirculation and gut perfusion. In a pragmatic way, following PAC and PDC would permit prediction of circulatory failure 20 minutes earlier. It supports the idea that this monitoring can be useful by anticipating macrocirculatory impairment. Stronger correlations on that setting may be due to different evolution of each signal after resuscitation. These results were consistent with previous studies carried out to understand the pathophysiology of sepsis [42,43].

We acknowledge some limitations to our study.

First, in opposite video microscopy, the tested probe is not conceived to evaluate heterogeneity of microcirculatory impairments, a condition described in sepsis [44].

As there is no existing device using PPG to monitor gut perfusion, we could not compare the probe to a reference apparatus. Even if existing data often suggest a strong link between PPG and LD, both techniques use different optical arrangements, which make a direct comparison of the signals produced difficult [45]. However, PDC and PAC decreased in a similar fashion early during sepsis and increased after resuscitation maneuvers, which tend to confirm that they monitor similar parameters.

It is quite hard to know what the PPG signal represents exactly. One can argue that it does not reflect the microcirculation, as it is a pulsatile signal, nevertheless LD, even if it is pulsatile, is often referenced as a tool to assess microcirculation. Microcirculation starts at the arteriolar level. The cutoff diameter is about 100 micrometers [19]. Pulsatility disappears near arteriolar level but could vary according to arteriolar stiffness [46]. Even if PAC seems more sensitive to hemodynamic change, the PDC part of the signal is not pulsatile and was also significantly correlated to GM and lactate.

We do not know the penetration depth of the signal exactly. According to a previous study, the depth could be 10 to 12 mm with similar sensor properties for size and wavelength; however, this study was performed on the skin and no data have been found concerning depth of the signal sampling in the gut [45]. The sampling volume of current LD devices is between 0.5 and 1 mm^3^[19]. Compared to these data, PPG seems to sample a larger part of the tissue and may also receive signals from the arteriolar level.

The experimental settings could have influence the microcirculation. Animals were anesthetized throughout the experiment, which was necessary to allow use of the equipment on the animals, blood sampling and accurate measurements. Nevertheless, general anesthesia is a confounding factor, because anesthetic drugs might have affected the microcirculation [47] in both groups. Surgery as well is known to alter the microcirculation [48]. The inflating balloon was a major concern as it could have affected the microcirculation of the duodenum. When using video microscopy, it is known that a little pressure on the mucosa can impact the measurement of microcirculation [19].

Nonetheless, we measured a PPG signal throughout the experiments under the same conditions. An important feature of the device is that it can adapt the inflation pressure according to intra-abdominal pressure (the balloon pressure is 2 kPa above the intraperitoneal pressure resulting in the same the transparietal pressure for every measurement), thus preventing any hyperinflation.

One could fear that inflating a balloon in the duodenum could create lesions such as ulcers or perforations, especially in a weakened intestinal wall. Nevertheless, the inflation pressure, 2 kPa is low in comparison with the pressure used by an enteroscope (a technique that inflates a balloon in the duodenum and the ileum up to 5.4 kPa for more than eight minutes). On the other hand, ischemic risk of balloon inflation is not supported by the histological results of our study.

Even though the device is designed to be introduced by mouth, we positioned the probe in an invasive fashion after gastrectomy, which led us to perform a surgery that could have influenced microcirculation. Nevertheless, we needed to perform a median laparotomy to place the LD probe and introducing the probe by gastrectomy allowed us to better standardize the equipment of the piglets, as we could better control the advancement of the probe in the duodenum.

One can argue that placement of a duodenal feeding tube in septic patients is not a standard practice. We must emphasize that a majority of patients in intensive care may need an enteral feeding tube during the time course of sepsis. In a population of intensive care unit patients in the UK, 54.6% received enteral feeding via nasogastric tube and only 1.5% received it via nasojejunal tube. Nasogastric tube is controversial [49] and we should consider that post-pyloric nutrition may reduce the risk of aspiration pneumonia, increases calorie intake and reduce the use of parenteral feeding [50]. The features of this new probe match the needs of patients in intensive care units in a noninvasive manner. Clinicians fear the placement difficulties of nasojejunal tubes but this statement is not supported by the literature. Ninety-two percent of successful placement has been reported [51]. If the patient needs enteral feeding, the choice of this new device brings the potential advantage of intra-abdominal pressure monitoring, post-pyloric feeding and a noninvasive monitoring of gut perfusion.

Finally, some technical details can be improved. The balloon pressure was set to record the best signal, but we may consider a different pressure or multiplying level of pressure to capture better microcirculation and perfusion results. Another wavelength could be added to assess gut oximetry at the same time. The distance between the light-emitting diode and photodetector could be reduced as different choices of wavelength could be made to reduce the sampling volume. Further investigation will address these points.

## Conclusions

Based on our results, we conclude that the PPG probe is able to detect an impairment of the gut perfusion in an experimental model of septic shock. As a need for a reliable means of microcirculation monitoring is mandatory, the new device evaluated in this experiment gives valuable information. A strong link with gut perfusion evaluated with the LD probe has been established, even though the nature of the signal needs to be better characterized. We think that the probe is worth further investigation and one day may help the clinician to set resuscitation goals on gut perfusion endpoints.

## Key messages

• This is the first evaluation of the evolution of gut photoplethysmographic signal composed of PAC and PDC during septic shock. We performed this study in the context of development of a new device.

• PAC and PDC are linked to the circulatory failure in the context of septic shock.

• A correlation of the PAC and PDC to the laser Doppler signal of the gut and lactate assays has been observed.

## Abbreviations

AU: arbitrary unit; CFU: colony-forming unit; CI: cardiac index; CO: cardiac output; GM: gut microcirculation; GM_var_: variation of the GM; HR: heart rate; LD: laser Doppler; MAP: mean arterial pressure; MPAP: mean pulmonary arterial pressure; PAC: AC part of the photoplethymographic signal; PAC_var_: variation of the PAC; PCWP: pulmonary capillary wedge pressure; PDC: DC part of the photoplethymographic signal; PDC_var_: variation of the PDC; PPG: photoplethysmography; RL: Ringer’s lactate; SPAP: systolic pulmonary arterial pressure; SPO2: oxygen saturation measured by pulse oximetry.

## Competing interests

The authors declare that they have no competing interests.

## Authors’ contributions

MJL drafted the manuscript and participated in the statistical analysis. MJL, CP, JYA, JMBG, OV, DR and SJ carried out the experimentation, data collection and resynchronization of the data and contributed to study design; they also revised the manuscript critically for important intellectual content. SJ designed the study and performed the statistical analysis. JMBG and BA contributed to the study design and coordination. BA also helped to draft the manuscript. CP, JYA, JMBG, OV, DR, SJ, JE, FV, and OD also revised the manuscript critically for important intellectual content. JE, FV and OD participated in the design and helped to draft the manuscript. All authors read and approved the final manuscript.
